# On the Medicinal Mixtures Employed as Styptics

**Published:** 1845-02

**Authors:** 


*On the Medicinal Mixtures employed as Styptics.*—By Dr. GOTTSCHALK.—Dr. Gottschalk thinks that a medicinal mixture can act as a styptic only when it is not applied in the liquid form. "He has demonstrated," he says, "that vegetable astringents do not merit this denomination, that they are not in reality astringents properly so called, because if they tan the tissues they give rise to a chemical combination which is accompanied by a thickening and not by a contraction of those tissues." The experimentalist has made several trials with portions of intestine and pieces of liver which he allowed to remain for five days in solutions of sulphate of copper, sulphate of zinc, sulphate of iron, acetate of lead, crystallised alum, calcined alum, sulphuric acid, hydrochloric acid, nitric acid, and creosote. The results which he obtained in these experiments led him to the following conclusions:—

1st. The strongest styptics, alum, acetate of lead, and sulphate of copper, lose their styptic virtue when they are employed in the liquid form.

2nd. The liquid form is opposed, on the other hand, to the contraction of the tissues; on the other hand, it gives rise to a softening of the animal substance, and, consequently, it facilitates imbibition, impregnation, the thickening, and enlargement of the tissues, and it thus diminishes the tendency to destructibility.

3rd. The acids employed, except nitric acid, do not possess any styptic property; but they possess that property of rendering the tissues thicker.

Dr. Gottschalk, in extending his investigations to the decoctions of oak, rhatany, tormentilla, and nutgalls, in which he steeped for eight days dried pieces of intestines, and for five days pieces of sclerotica, cornea and conjunctiva of the ox in the fresh state, arrived at the following conclusions:—

1. None of the astringents indicated merit this name when they are employed under a form which prevents them from removing water from the tissues which are found in contact with them.

2. They are so much the less astringent, as in the liquid form they penetrate deeply into the tissues, and as they consequently produce thickening and enlargement.

3. If we omit the principles which, like strychnia, determine contraction, in consequence of their action on the nervous system, there remain as agents of styptic medication only the medicines called exsicants and refrigerants.—*Chemist.*

